# Study on instability mechanism of soft rock roadway and pressure-relief bolt-grouting support technology

**DOI:** 10.1038/s41598-023-47451-8

**Published:** 2023-11-24

**Authors:** Tuo Yang, Jianzhuang Liu, Jianqiao Luo, Yupeng Shen, Peng Fu

**Affiliations:** 1 School of Energy and Mining Engineering, China University of Mining and Technology(Beijing), Beijing, 100083 China; 2 School of Mining Engineering, North China University of Science and Technology, Tangshan, 063210 Hebei China

**Keywords:** Energy science and technology, Engineering

## Abstract

Aiming at the engineering problem of roadway deformation and instability of swelling soft rock widely existed in Kailuan mining area, the mineral composition and microstructure of such soft rock were obtained by conducting scanning electron microscopy, X-ray diffraction experiments, uniaxial and conventional triaxial tests, and the law of softening and expanding of such soft rock and the failure mechanism of surrounding rock were identified. The combined support scheme of multi-level anchor bolt, bottom corner pressure relief and fractional grouting is proposed. The roadway supporting parameters are adjusted and optimized by FLAC3D numerical simulation, and three supporting methods of multi-layer anchor bolt, bottom corner pressure relief and fractional grouting are determined and their parameters are optimized. The study results show that: the total amount of clay minerals is 53–75%, pores, fissures, nanoscale and micron layer gaps are developed, providing a penetrating channel for water infiltration to soften the surrounding rock; the three-level anchor pressure-relief and grouting support technology can control the sinking amount of the roof within 170 mm, the bottom drum amount within 210 mm, the bolts of each level is evenly distributed in tension, and the maximum stress and bottom drum displacement in the pressure relief area are significantly reduced; the pressure-relief groove promotes the development of bottom corner cracks, accelerates the secondary distribution of peripheral stress, and weakens the effect of high stress on the shallow area. Using time or displacement as the index, optimizing the grouting time, filling the primary and excavation cracks, blocking the expansion and softening effect of water on the rock mass, realizing the dynamic unity of structural yielding pressure and surrounding rock modification, has guiding significance for the support control of soft rock roadway.

## Introduction

For a long time, the difficulty of supporting soft rock roadway has been puzzling the mining convergence and safety production. With the depletion of shallow resources, mining activities gradually move to the deep. The deep roadway has typical characteristics such as high original rock stress, high-pressure karst water, multiple geological structures and complex hydrological conditions. In addition, the roadway section tends to increase under the requirements of ventilation, transportation and reservation deformation, leading to great challenges for roadway support technology. Anchorage instability, support deformation and roof collapse occur from time to time^1–3^, which seriously affects the mining and connecting plan and brings great losses to coal mine production.

The mineral composition of soft rock mainly includes clay minerals, quartz and other minerals. The common clay minerals include kaolinite, montmorillonite, illite, chlorite and illite/montmorillonite mixed layer. Due to the special characteristics of clay minerals easy weathering and water swelling, the stability of roadway is often affected. Due to unloading, weathering, especially the influence of water, surrounding rock will produce hydration expansion, strength reduction, softening collapse and even slime, which will induce engineering disasters such as roof collapse, floor heave and even collapse of soft rock roadway^4–6^.

In response to the above technical difficulties, relevant institutions have carried out a large number of theoretical derivation, experimental testing, mine pressure monitoring, support component upgrading, engineering practice optimization and other work^7–10^. Based on the new Austrian method, combined support technology, primary and secondary bearing area support theory, stress control theory and other theories, new technologies such as coupling support, strength strengthening of surrounding rock, hysteretic grouting reinforcement, geomechanical testing of original rock, and support technology of prestressed anchor bolt have been formed^11–14^. New methods such as concrete filled steel tube, shed-cable integrated support, anchor cable with constant resistance and large deformation, combined grouting, and prestressed steel rod have been explored. Recently, new control methods such as portal support, full-section anchor cable, and ultra-high strength anchor bolt have appeared^15–17^.

He^18^ established the process function of mudstone's water absorption based on the water absorption tests of different mudstone samples, and analyzed the influence rule of mudstone porosity size, mineral content and type, and occurrence of clay minerals on water absorption characteristics. Lian^19^ conducted natural water absorption tests on soft kaolinite blocks and found that the water absorption rate of soft kaolinite blocks increased negatively with the increase of soaking time. Liu^20^ studied the effect of water saturation on the strength parameters, deformation characteristics and energy evolution of mudstone. Fan^21^ studied the saturation variation law of mudstone during the process of water-soaked expansion and water-loss shrinkage through laboratory expansion and shrinkage tests, and analyzed the saturation variation law and expansion deformation failure mechanism combined with the suction and water potential change theory. Chai^22^ applied the Grand Canonical Ensemble Monte Carlo (GCMC) and molecular dynamics (MD) methods to study the water absorption characteristics of kaolinite supercell model, and obtained the adsorption capacity, adsorption site, adsorption heat and adsorption energy of water molecules on kaolinite surface at different temperatures and pressures, as well as the volume expansibility of kaolinite after water absorption. The molecular mechanism of the interaction between kaolinite and water molecules was revealed. Liu^23^ conducted an experimental study on the swelling behavior of bentonite with different montmorillonite contents and found that all indexes of the swelling behavior of formed bentonite (axial maximum swelling force and radial maximum swelling force) increased with the increase of montmorillonite content in a linear relationship.

He^24,25^ effectively controlled the large deformation problem of soft rock roadway in Shaji mine by adopting the method of constant resistance device + steel mesh + bottom corner grouting anchor; Wang^26,27^ improved the overall bearing capacity of soft rock roadway supporting structure by anchoring method; Bai^28,29^ proposed the active pressure relief + grouting scheme in Guhanshan Mine. The expansion deformation of surrounding rock can be effectively released and the integrity and strength of surrounding rock can be improved. Zhang^30^ analyzed the main reasons for the poor grouting effect of roadway in Zhuji Coal Mine and optimized the hysteretic grouting parameters of roadway surrounding rock. Chen^31^ maintained the stability of track roadway in Cheji Mining Area 28 by opening a pressure relief groove in the floor and carrying out secondary reinforcement support. To prevent large deformations of deep soft rock roadways, Guo^32^ proposed a combined support technology consisting of constant resistance large deformation bolts, steel strips, and grouting bolt pipes. Qi^33^ proposed the bearing strength theory of a superimposed arch under the condition of “anchor spray net + anchor cable” combined support for deep weak surrounding rock. Zhang^34^ pointed that the range and shape of the plastic zone of the surrounding rock were the theoretical basis for the quantitative design of the supporting structure and proposed a support scheme for weak cemented soft rock roadways. Wang^35^ studied the deformation characteristics of surrounding rock of deep soft rock roadway, and found that the main reason for the failure of soft rock roadway was unreasonable support scheme and improper support form, which could not withstand the deformation of soft rock roadway, and proposed a bearing concrete support scheme to maintain the stability of roadway. Kang^36^ proposed that the use of ultra-high bolts with high initial anchoring force supplemented by grouting and energy release measures can maintain the stability of the deep roadway. Zhang^37^ studied the bearing mechanism of the double bearing structures in the deep soft rock roadway and regarded the surrounding rock of the broken roadway as a small supporting structure and the deep surrounding rock as a large bearing structure.

The above technology can not be widely applied to soft rock roadway under various geological conditions. The supporting strength is generally 1–2 MPa, and the adjustment range is far less than the difficulty caused by mining depth, structure and lithology softening. In addition, the economic cost of upgrading the supporting density and strength is large, which limits the selection of related measures such as anchor bolt encryption, heavy support, concrete pouring and floor heave anchoring. Therefore, the theoretical basis and technical selection of soft rock support technology still need to be based on the specific geological environment, grasp the deformation law, check the instability mechanism, in order to achieve the scientific selection and reasonable matching of anchoring, unloading, injection and other technical means, so as to achieve economic and effective roadway support.

The soft rock support in Kailuan mining area has been restricting the development of deep resources. Especially in recent years, the whole mining area has entered deep mining, and the geomechanical environment is extremely complex. The difficulty of support has increased sharply. It is urgent to study the deformation mechanism and stable support technology of roadway.

This paper takes the roadway engineering of surrounding rock rich in montmorillonite as the background, starting from the micro aspect, probes into the micro-morphology and mineral composition and content of surrounding rock, analyzes its mechanical properties through mechanical tests, technically addresses the deformation and instability mechanism of roadway, and explores the stable support mode and dynamic construction method. The practice of optimizing the combined support scheme of pressure-relief + bolt-grouting will provide important technical reference for the safe construction and sustainable development of the whole mining area and other roadway rich in montmorillonite at home and abroad, which is of great significance.

## Project overview

The strata of some roadways in Kailuan mining area are characterized by brittle lithologic and fissure development, mainly composed of gray or grayish-white argillaceous sandstone, which has strong expansibility and high content of clay minerals, and belongs to typical softened and swelling surrounding rock. It is revealed in the construction of some roadways that it is greatly influenced by fault fissure aquifer and frequent structural cutting. The roof sandstone fissure water guided by fissure excavation along the primary structural fissure and roadway leads to water softening and expansion of surrounding rock. The deformation speed of roadways is fast after initial excavation, and the deformation becomes unstable after 15~20 days, which leads to the failure of driving in later period. The softening rheological property of roadway is obvious.

## Microscopic characteristics and macroscopic deformation analysis

### Sample profile

Common clay minerals in the construction of underground soft rock roadway include montmorillonite, kaolinite, illite and chlorite, and their morphological characteristics are shown in Table 1.

**Table 1:** Morphological characteristics of several common clay minerals.

Types of clay minerals	Color	Monomer morphology	Common aggregate form
Montmorillonite	White gray, light red	Fine scale, petaloid	Honeycomb, felt, flocculent
Kaolinite	White, red, blue, brown	False hexagonal flake, flake	Earthy, dense and massive
Illite	White, yellow, green, brown	Scaly, striate	Soil-like, slate-like
Chlorite	Light green to green black	Lamellar or platelike	Scaly, rose-petal, earthy

**Figure 1:**
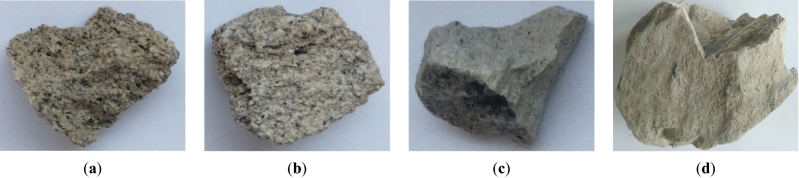
Apparent characteristics of rock samples. (**a**) 1# rock sample, (**b**) 3# rock sample, (**c**) 4# rock sample, (**d**) 6# rock sample.

The apparent characteristics are shown in Fig. 1. In the process of sample processing, it is found that the rock sample # 1 and # 3 are gray and white coarse-grained sandstone with small hardness, which can be crushed by hand. Rock sample 4# is gray-green medium-grained sandstone, and rock sample 6# is gray-white medium-grained sandstone, with small hardness and obvious cutting edge.

### Analysis of mineral composition in surrounding rock

**Figure 2:**
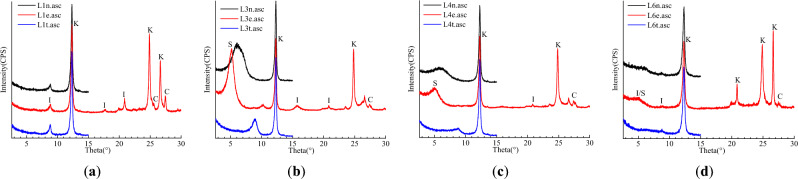
Diffraction pattern of rock sample. (**a**) 1# rock sample, (**b**) 3# rock sample, (**c**) 4# rock sample, (**d**) 6# rock sample.

In order to explore the softening and swelling mechanism of surrounding rock from the aspects of microstructure and mineral composition, Quanta°250° environmental scanning electron microscope system (FEI) and D/MAX-rA X-ray diffractometer were used to observe the morphology of rock samples and determine the mineral composition. The experimental conditions were 23℃ at room temperature and 62% humidity. According to the X-ray diffraction analysis method (SY/T 5163-2010) for clay minerals and common non-clay minerals in sedimentary rocks, the method is to use the preparation of directional plates (natural directional plates, ethanol saturated plates, high temperature plates) for qualitative and quantitative analysis. The diffraction pattern curve of the samples was obtained, as shown in Fig. 2. Compared with the characteristic values of the standard diffraction pattern, the composition content of clay minerals in each sample was obtained through phase analysis, as shown in Table 2.

**Table 2:** Composition content of clay minerals.

Sample	Apparent description	Clay mineral content (%)	Relative content of clay minerals (%)					Mixed layer ratio (%)
			S	I/S	I	K	C	I/S
1#	Gray-white coarse sandstone	75	–	–	16	76	8	–
3#	Gray-green medium sandstone	53	24	–	1	72	3	–
4#	Gray coarse sandstone	70	47	–	1	48	4	–
6#	Gray-white medium sandstone	55	–	10	1	87	2	50

Remarks: *S* montmorillonite, *I/S* illite/montmorillonite mixed layer, *I* illite, *K* kaolinite, *C* chlorite, *C/S* chlorite/montmorillonite mixed layer.

As can be seen from the chart, the content of clay minerals is about 53–75%, and the mineral types are mainly mixed layers of kaolinite, montmorillonite and I/S. The 1# rock sample is mainly kaolinite, a small amount of illite and chlorite, and the content of kaolinite is 76%. The 3# rock sample is mainly kaolinite and montmorillonite. The 4# rock sample is about 50% of montmorillonite and kaolinite. The 6# sample is mainly kaolinite, and the content of I/S is 50%. Montmorillonite has strong water absorption characteristics, and its water absorption rate can reach 20%-80%. Kaolinite has strong water absorption and softening property, which is the material basis for the hydration instability of surrounding rock^38^.

### Analysis of surrounding rock microstructure

**Figure 3:**
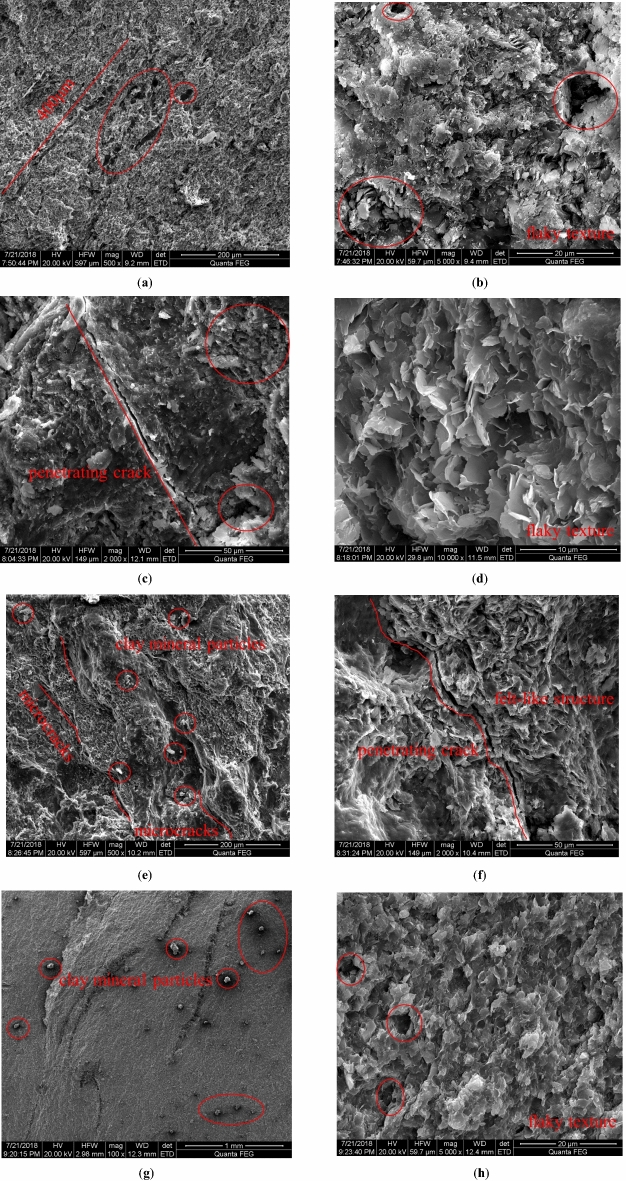
SEM photos of rock samples. (**a**) Sample 1# was magnified 500 times, (**b**) sample 1# was magnified 5000 times, (**c**) sample 3# was magnified 2000 times, (**d**) sample 3# was magnified 10,000 times, (**e**) sample 4# was magnified 500 times, (**f**) sample 4# was magnified 2000 times, (**g**) sample 6# was magnified 100 times, (**h**) sample 6# was magnified 5000 times.

In Fig. 3a, b, the SEM photo of sample 1# showed loose lithology in general appearance. When magnified 500 times, a large number of cracks could be seen, and pore development was extremely obvious, including nanoscale micropores, transition pores and mesoporous pores. When magnified 5000 times, a large number of irregular intergranular pores could be found, and complex intergranular pores were formed inside, and the aggregate was irregular flake.

In Fig. 3c, d, the SEM photo of sample 3# showed that there were gray and white clay minerals on the surface when enlarged by 2000 times, and the minerals were distributed in granular and flaky forms, with penetrating cracks and a large number of micro-cracks, and the pores were obviously developed. When enlarged by 10,000 times, it could be seen that the scaly structure and interlayer cracks were extremely developed, the petal-like laminated structure was very obvious, and the micron-scale pores were the most developed.

In Fig. 3e, f, the SEM photo of sample 4# showed loose lithology in general view. When magnified 500 times, the surface was rich in clay minerals, with relatively developed microfractures and pores including nano-scale micropores, transition pores and mesopores, forming irregular intergranular pores. There were a large number of complex mesh intergranular pores inside, and the aggregate was felt like.

In Fig. 3g, h, the SEM photo of sample 6# showed that a large number of grayish white clay mineral particles were distributed on the surface when magnified 100 times, and there were insignificant micro-cracks. When magnification was 5000 times, the lamellae structure was very obvious, and the micron-scale pores are the most developed and the pores were obviously developed, minerals were distributed in granular form.

Through SEM photos, it could be found that there was a lack of chemical cement among the particles in this kind of surrounding rock, and the pores were connected to each other in the deep, which provides a smooth space channel for the infiltration of crack water in the roof sandstone. It could be seen that this kind of surrounding rock had strong water absorption, and the process of hydrolytic softening and expansion was fast, which was the main mechanism of the softening and support instability of roadway surrounding rock.

### Softening and swelling mechanism of surrounding rock

It was found that the content of clay minerals was up to 53–75%, and the clay minerals were mainly kaolinite and montmorillonite. The closer the color was to grayish white, the content of montmorillonite was higher. Montmorillonite crystals belong to aquifer-like silicate minerals of monoclinic crystal system, generally composed of two layers of silico-oxygen tetrahedrons sandwiched by one layer of alumino-oxygen octahedrons, belonging to the 2:1 layered minerals, whose structural formula is Al_2_(Si_4_O_10_) (OH)_2_·nH_2_O, with fine particles, about 0.2–1 μm, weak interlayer connection, and colloidal dispersion characteristics. There is water and some exchange cations between the crystal structural layers, and there is a high ion exchange capacity, which causes the crystal layer to expand, so it dissolves quickly after encountering water. It disintegrates quickly upon contact with water.

The lattice structure of kaolinite is composed of a layer of silico-oxygen tetrahedral wafers and a layer of alumino-oxygen (hydroxyl) octahedral wafers. It is a 1:1 layered mineral. The theoretical structural formula is Al_4_(Si_4_O_10_) (OH)_8_. Chai^22^ found that the water absorption characteristics of kaolinite are very sensitive to the change of pressure, and the adsorption capacity increases logarithmically with the increase of pressure. Therefore, when the soft rock roadway rich in kaolinite is located in deep high-stress environment, it also has strong water absorption characteristics.

Soft rock minerals have strong hydrophilicity and high water absorption and expansion capacity. When the content of montmorillonite is greater than 30%, its saturated water absorption rate is more than 50%, and its free expansion deformation is more than 15%^39^. Such a strong water absorption capacity indicates that it has a great expansibility, which can expand and exceed the original volume 5–9 times. This is also the reason why this kind of soft rock expands and deforms to different degrees after encountering water.

Under the action of deep excavation stress, the microfissure in the surrounding rock is expanded and connected after being stressed. Water molecules exist between small flakes through the microfissure and make them expand. Water molecules also enter into the interlayer of the crystal cell, leading to internal expansion. In addition, they include external expansion, in which polarized water molecules enter between layered silicate clay mineral particles and cause them to expand, and stress dilatation. The latter is the volume expansion caused by the expansion and penetration of microfissure under the force of rock mass. It can be seen that internal expansion and external expansion mechanism is a physicochemical mechanism under the action of rock and water, while stress dilatancy expansion is a mechanical mechanism. The expansion deformation in actual soft rock engineering is often caused by the combined action of three expansion mechanisms.

### Macro analysis of surrounding rock deformation

**Figure 4:**
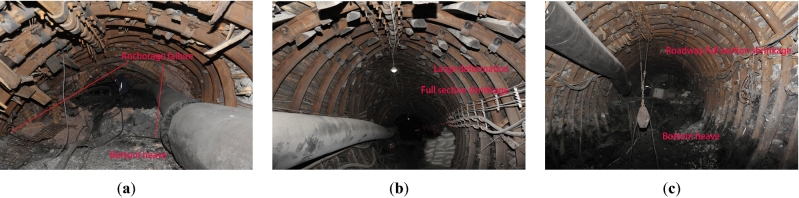
Deformation of local roadway. (**a**) Anchorage failure. (**b**) Full section shrinkage. (**c**) Bottom heave.

With large deformation in the whole section of the roadway and short stabilization time, long-term rheologies with observable characteristics exist. With sufficient cracks in the shallow surrounding rock and expansion of the broken zone, the anchorage force attenuates and the low resistance failure of the bolt in a large range, as shown in Fig. 4a, the structural support of the anchorage zone is eventually lost. The deformation characteristics of the U-shaped steel shed support are large deformation and full section shrinkage, as shown in Fig. 4b,c. The reason is that there is a large amount of water in the sandstone fissure in the roadway roof, and the primary fissure and excavated fissure in the surrounding rock run through each other, providing a large number of channels for water infiltration and drip seepage flow, leading to the hydrolytic expansion and softening of the surrounding rock, the weakening and instability of the original anchor structure, and the support of the roadway will bear a high pressure of the surrounding rock, and finally breaking through the stability strength of the system will lead to the instability. Roadway renovation reveals that the broken zone of surrounding rock mostly exceeds the root of the anchor bolt, and there is no mass breaking phenomenon of the anchor bolt. Therefore, the key point of support strengthening will be the adjustment of the length of the anchor bolt and the improvement of the anchoring performance.

## Compressive test study

Due to the limitation of field sampling conditions and the need to pay attention to key strata, only the gray white medium sandstone commonly existed in Kailuan mining area was measured. The preparation process and test process of the specimen were strictly in accordance with the "Engineering Test Method Standard for Rock Mass" and the "Suggestions and Methods for Rock Mechanics Tests" compiled by the Committee for Standardization of Laboratory and Field Experiments of the International Society of Rock Mechanics. The diameter of the specimen should be 49–50 mm and the height should be 100–101 mm. The processing accuracy of the specimen should refer to the above standards.

Uniaxial and conventional triaxial compression tests were carried out by TAW-2000 electro-hydraulic servo triaxial rock testing machine. Two kinds of dry samples and saturated samples were measured in total. The samples with homogeneous apparent characteristics were selected and placed in the oven for 24 h at 105–110 ℃, and then cooled to room temperature in the oven to prepare the dry samples required for the test. The saturated sample was prepared by free suction method and soaked in mine water for 48h. Confining pressure Six groups between 0 and 25 MPa were selected according to the in-situ stress measurement results.

**Figure 5:**
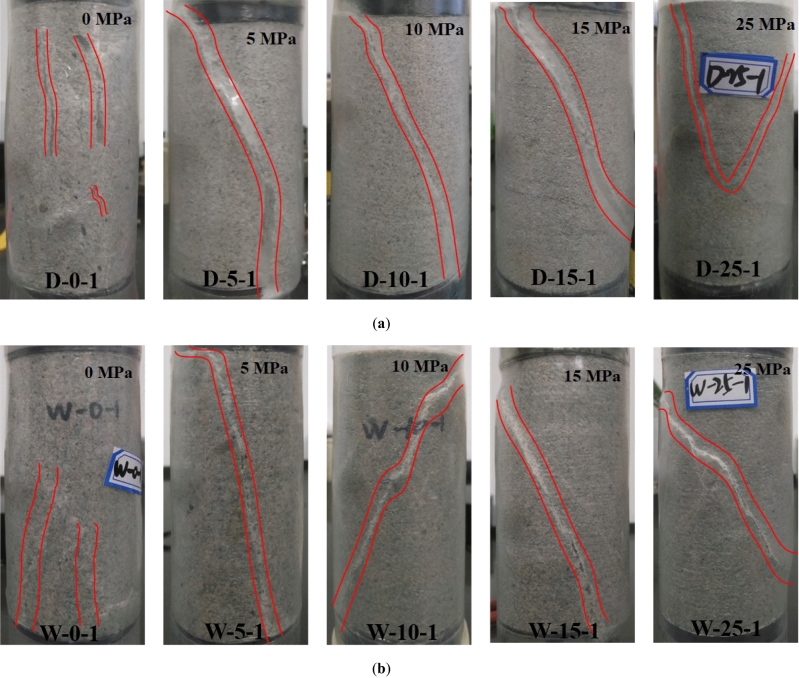
Failure mode of rock sample experiment. (**a**) Dry rock sample, (**b**) wet rock sample.

In Fig. 5, under uniaxial compression, the failure mode of both wet and dry rock samples was vertical split crack, which indicated that the leading mode of surrounding rock instability was tensile failure. Under the confining pressure of 5–25 MPa, the rock failure showed a single crack in oblique shear or a cone crack in opposite top, which indicated that the leading mode of rock instability under this condition was Coulomb shear failure.

**Figure 6:**
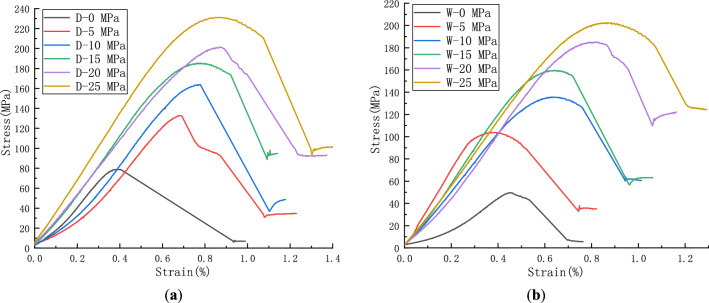
Triaxial test results of rock samples. (**a**) Dry rock sample. (**b**) Wet rock sample.

In Fig. 6, the uniaxial compressive strength of dry rock samples was 78.8 MPa. With the increase of confining pressure, the compressive strength gradually increases to 231.1 MPa, and the ultimate axial strain above 5 MPa confining pressure ranges from 0.7 to 0.8%. The uniaxial compressive strength of wet rock samples was 49.7 MPa. With the increase of confining pressure, the compressive strength gradually increases to 202.6 MPa, and the ultimate axial strain above 5 MPa confining pressure ranges from 0.4 to 0.9%. It can be seen that dry rock samples have high bearing capacity under triaxial compression. With the confining pressure increasing to 25 MPa, the ultimate stress can reach more than 200 MPa. The softening effect of water on specimens with low confining pressure was extremely significant, and the softening coefficient of uniaxial compressive strength was 63.1% (less than 75%), indicating poor softening resistance. The axial strain curve had an obvious post-peak drop section in the late plastic period, and the residual strength increases with the increase of confining pressure.

Corresponding to underground engineering, due to the exposure of small faults and abundant water filling in roadway construction, the surrounding rock has been soaked by mine water for a long time, resulting in the objective existence of lithologic softening. According to the stress–strain curve obtained by the test, the results of uniaxial and triaxial tests under different confining pressures are compared. It is found that the strain in the plastic zone of surrounding rock is obvious, the residual stress of the rock under low confining pressure is low, and the post-peak bearing capacity is poor. For engineering, once the surrounding rock exceeds the plastic strain limit, the loose zone of surrounding rock will develop rapidly. It can be seen that deep infiltration or immersion of rock mass by water must be blocked timely and effectively, and timely grouting of loose and broken area to re-consolidate broken surrounding rock and improve its residual strength will be two important technical means to promote the self-stability of surrounding rock in roadway plastic zone and broken zone.

## Optimization of soft rock roadway support technology

### Roadway deformation control countermeasures

Based on the analysis of mineral composition, microstructure and physical and mechanical properties of gray-white sandstone, the surrounding rock of Kailuan mining area is rich in clay minerals, the strong support strategy of multi-level anchor unloading was determined. The anchor solid formed by multi-level dense anchor bolt was taken as the main supporting load, and a pressure relief trough was dug at the bottom corner of the roadway to achieve rapid pressure relief and crack stimulation after initial excavation. To provide a slurry expansion channel for the following two-level grouting, consolidate the shallow surrounding rock fissure with pressure after the peak in time, effectively block the deep infiltration or soaking of water to the rock mass, and rebond the partially failed anchor bolt to realize the reinforced bearing capacity in the 2.0–2.4 m anchoring zone and ensure the self-stability of surrounding rock in the plastic zone and broken zone of roadway.

**Figure 7:**
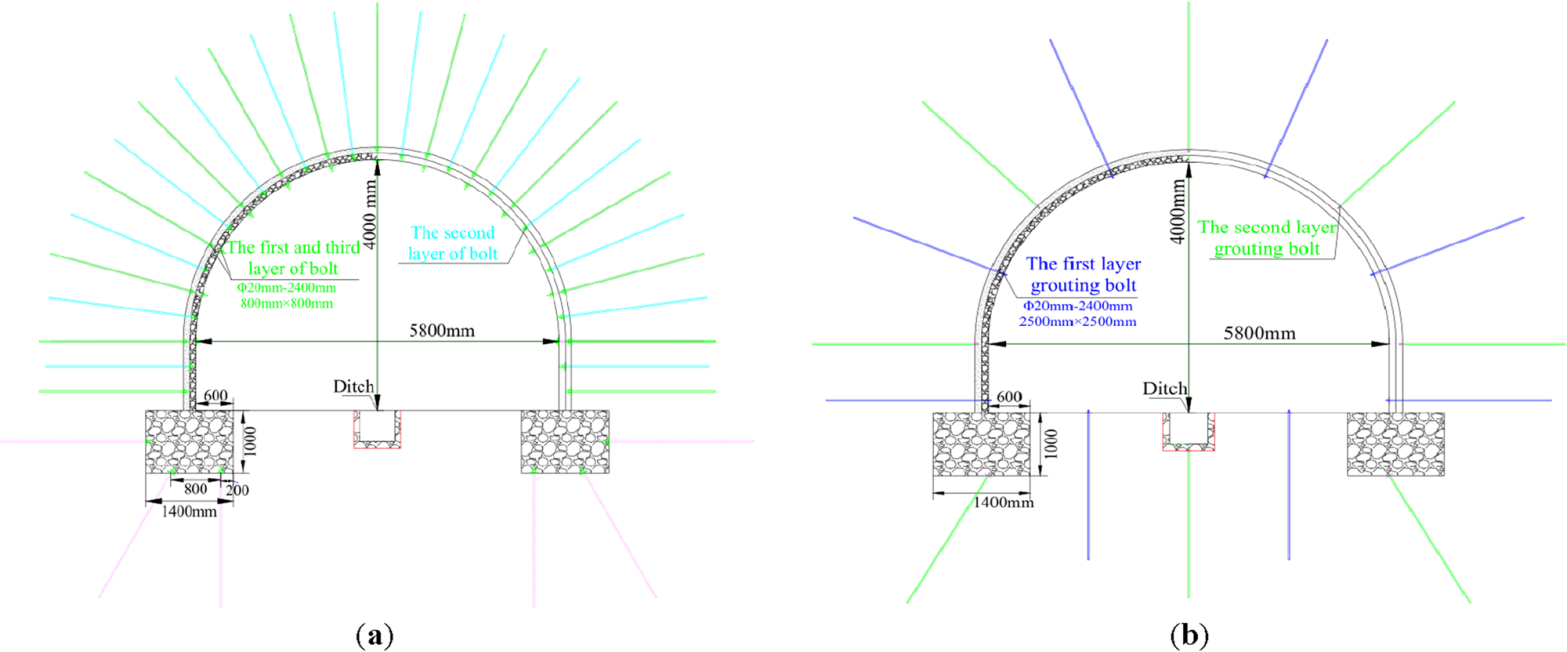
Multi-level anchor unloading support. (**a**) Three-layer rebar steel bolt. (**b**) Two-layer grouting bolt.

According to the far field stress, crack development and water seepage of surrounding rock, the specific parameters were designed as shown in Fig. 7. The deformation of roadway section was reserved for 200–400 mm, and the bolt specification was Φ20–2400 mm, and the row spacing between the first layer was 800 mm × 800 mm. Real-time monitoring of roadway deformation development was conducted. Every 100 mm convergence displacement of roof, floor or side can implement the next level of bolt support and set it in the upper neutral space. After the completion of the second level of anchor bolt, the excavation of pressure relief groove was carried out, and 3 bolts were installed for each pressure relief groove, at the same time, the first layer of grouting anchor bolts Φ20–2400 mm were installed, row spacing was 2500 mm × 2500 mm, 6 bolts were installed at the top of the roadway, and 3 bolts were installed at the bottom. The timing of surface convergence displacement of 100 mm was taken as the index to start the secondary high-pressure grouting. The top side of the second grouting bolt was 5, and each pressure relief groove was 1. The cement label of the two grouting was P.O42.5, the water-cement ratio was 0.7–1.1, and the grouting pressure was 1.5–2.5 MPa.

### Simulation and verification of roadway supporting parameters

**Figure 8:**
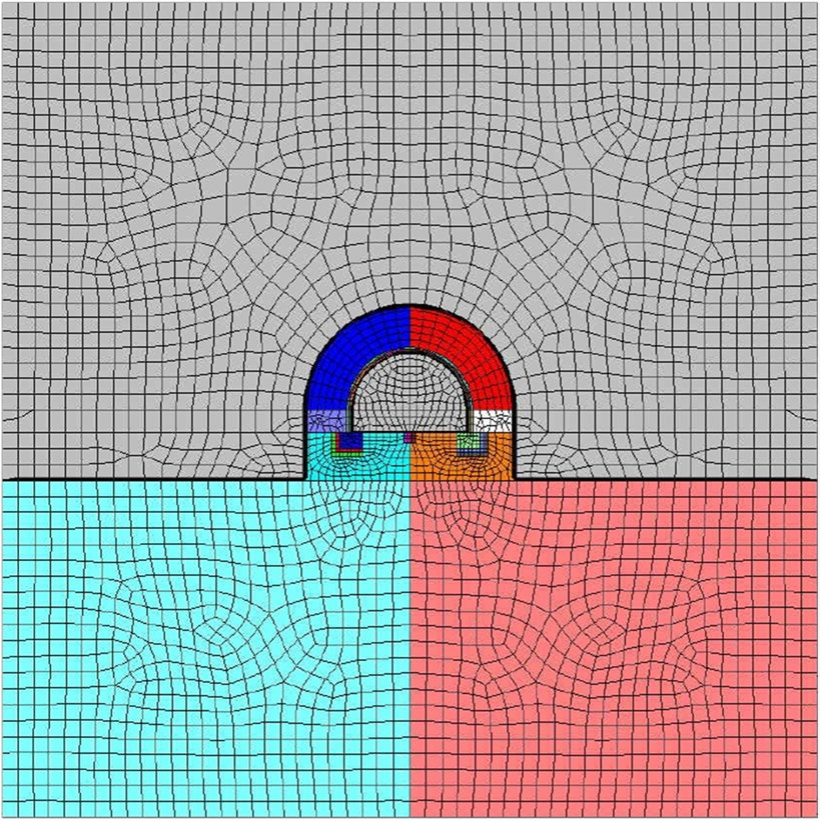
Pressure-relief bolt-grouting support model of roadway surrounding rock with rich montmorillonite.

In order to verify the rationality of the supporting parameters in Fig. 7, ANSYS12.1 was used to model object units and nodes, and then the model as shown in Fig. 8 was imported into FLAC3D to carry out the simulation calculation of loading conditions, boundary conditions and supporting parameters. The in-situ stress measurement data of -850 level soft rock roadway area in Kailuan Mining area were selected as the loading conditions. The lithologic parameters were calculated using CKW of the saturated rock sample in "Softening and swelling mechanism of surrounding rock", the elastic modulus was 26.7GPa and poisson's ratio was 0.275. The softening parameters included internal friction corner, cohesion, tensile strength and dilatancy corner, and the softening plastic strain boundary value was 15.0e−3.

**Figure 9:**
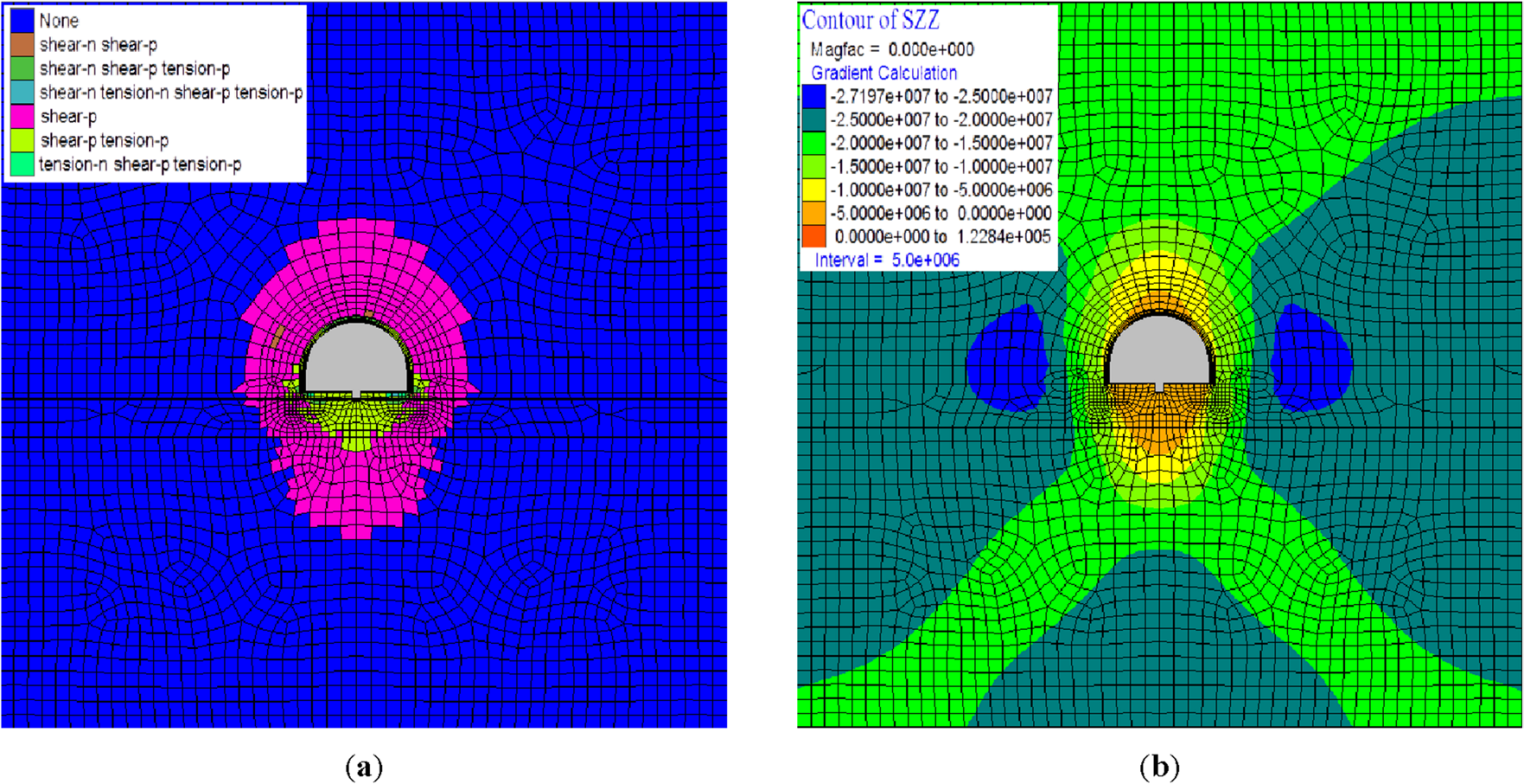
Simulation results of multi-level anchor unloading. (**a**) Plastic zone distribution and displacement. (**b**) Vertical stress σ_zz_.

Figure 9 shows the simulation results of multi-level pressure-relief bolt-grouting support. The thickness of plastic zone at the top is about 5.0 m, the thickness of plastic zone at the bottom is 5.8 m, and the thickness of plastic zone at the side is 2.6 m. Tensile failure zone and shear failure zone with a depth of 2.8 m appear in the bottom plate. The maximum vertical stress of 27.197 MPa is generated in the periphery of the two-sided plastic zone. The tension distribution of the three layers of bolt is more uniform, reaching the design load of 150 kN. The late displacement becomes stable with the calculation step, which can determine the stability of the support.

By adopting multi-layer grouting modification, the physicochemical action of water on swelling soft rock is well blocked, and the post-peak residual strength of shallow roadway surrounding rock is significantly improved, which is successfully applied in the first construction and first renovation of roadway in typical areas of Kailuan mining area. Using the cross section method, the measuring points were arranged in the middle of the roof and floor of the roadway, and the observation frequency was once every 2~3 days. After 120 days of the surface convergence and displacement observation of the roadway, it was found that the maximum convergence of the roof and floor of the roadway was 100 mm and the convergence rate was stable at 0.8 mm/day, and the maximum convergence rate of the two sides was 87 mm and the convergence rate was stable at 0.6 mm/day. After excavating the pressure relief groove at the two bottom corners of the roadway, the surrounding rock stress originally concentrated in the two bottom corners of the roadway is induced to transform into the deep part of the rock mass, forming an X-shaped conjugate stress reduction area. Compared to the no pressure relief groove scheme, the excavation of the pressure relief groove at the bottom corner of the roadway reduces the range of the surrounding rock stress concentration area and significantly weakens the degree of deformation at the bottom of the roadway, such as bottom heave. Combined with the anchor rod and grouting support scheme, the displacement and stress concentration phenomenon of the roadway surrounding rock are effectively controlled.

### Pressure relief mechanism experiment of small scale specimen

In order to study the stress transfer and deformation control mechanism of bottom corner pressure relief, based on the principle of physical similarity, 150 mm × 150 mm × 150 mm small scale rock-like material similar simulation roadway test blocks were poured by adding appropriate bentonite, quartz sand, barite powder and rosin alcohol solution with model gypsum as the base material. The biaxial loading experiment of the pressure relief mechanism was carried out, and the INAGE-IR8300 infrared thermal imager was used to monitor the energy activities around the roadway. The scale of the specimen was 1:150, and the prototype section of the roadway was 5.8 × 4.4 m semi-circular arch roadway. The 1# specimen had no pressure relief groove, and the size of the 2# specimen's pressure relief groove was 1.2 × 1.6 m.

**Figure 10:**
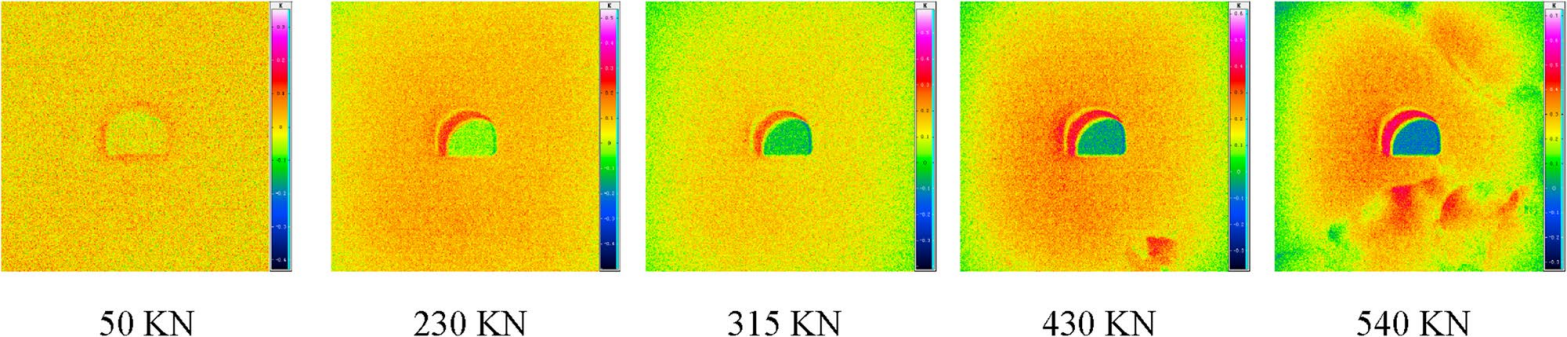
Thermal imaging monitoring of 1# specimen.

**Figure 11:**
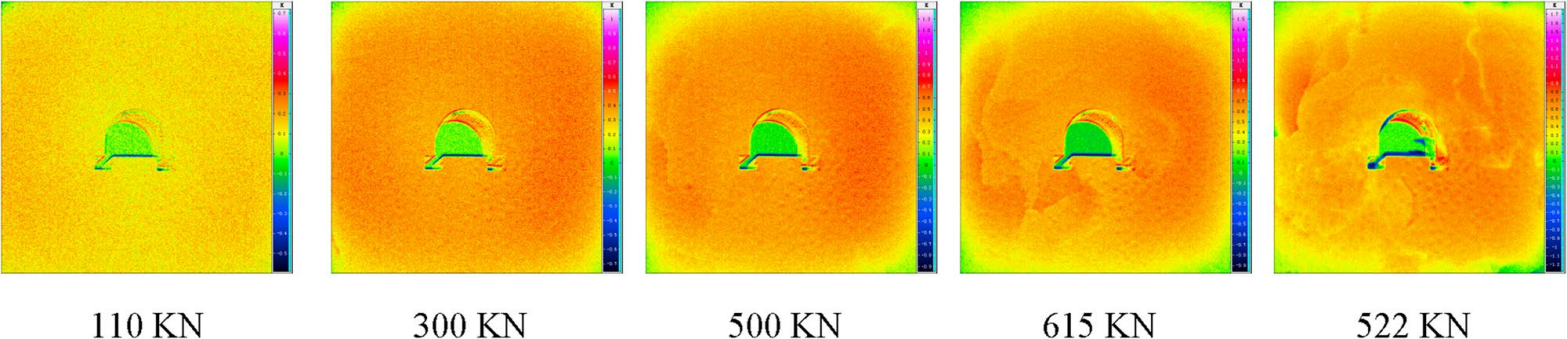
Thermal imaging monitoring of 2# specimen.

Figures 10 and 11 compare the surface temperature changes of specimens 1# and 2# until failure during loading. It is found that the temperature rise inside the roadway after loading is higher than that on the outside surface, indicating that large energy activities occur in the superficial area after the roadway is compressed. When 1# specimen was loaded to the axial stress of 50KN, it could be seen that energy activity occurred around the roadway. In the loading process, there was always a large energy activity inside the roadway until the failure. When the axial stress of specimen No. 2 was loaded to 110KN, the energy activity around the roadway began to appear. During the loading process, the energy activity in the shallow area inside the roadway was weak, and there was an obvious energy accumulation area at the bottom corner of the pressure relief groove, indicating that the relief groove promoted the development of the bottom corner crack, accelerated the secondary distribution of the peripheral stress, and weakened the effect of high stress on the shallow area.

### Field application of optimized support scheme

The engineering quantity of Qianjiaying Mine -850 main crosscut is about 1468 m. The surrounding rock is mostly gray and gray sandstone, rich in clay minerals, and the roadway surface is mudding when encountering water, which is easy to weather. The design section width of the roadway is 5.4 m and the height is 4.5 m. In the initial construction, conventional anchor net spray was used to support, and the pressure relief groove was excavated at two bottom corners of the chamber floor. The depth and width of the left bottom corner pressure relief groove is 800 × 1200 mm (inner wall width is 800 mm), and the right bottom corner pressure relief groove is 1000 × 1400 mm (inner wall width is 800 mm). According to the field exploration, the dynamic construction sequence is optimized: first-level anchoring → first-level grouting anchor → secondary anchoring → excavation of pressure relief groove → first-level grouting → backfilling of bottom corner pressure relief groove → installation of second-level grouting anchor → grouting reinforcement.

**Figure 12:**
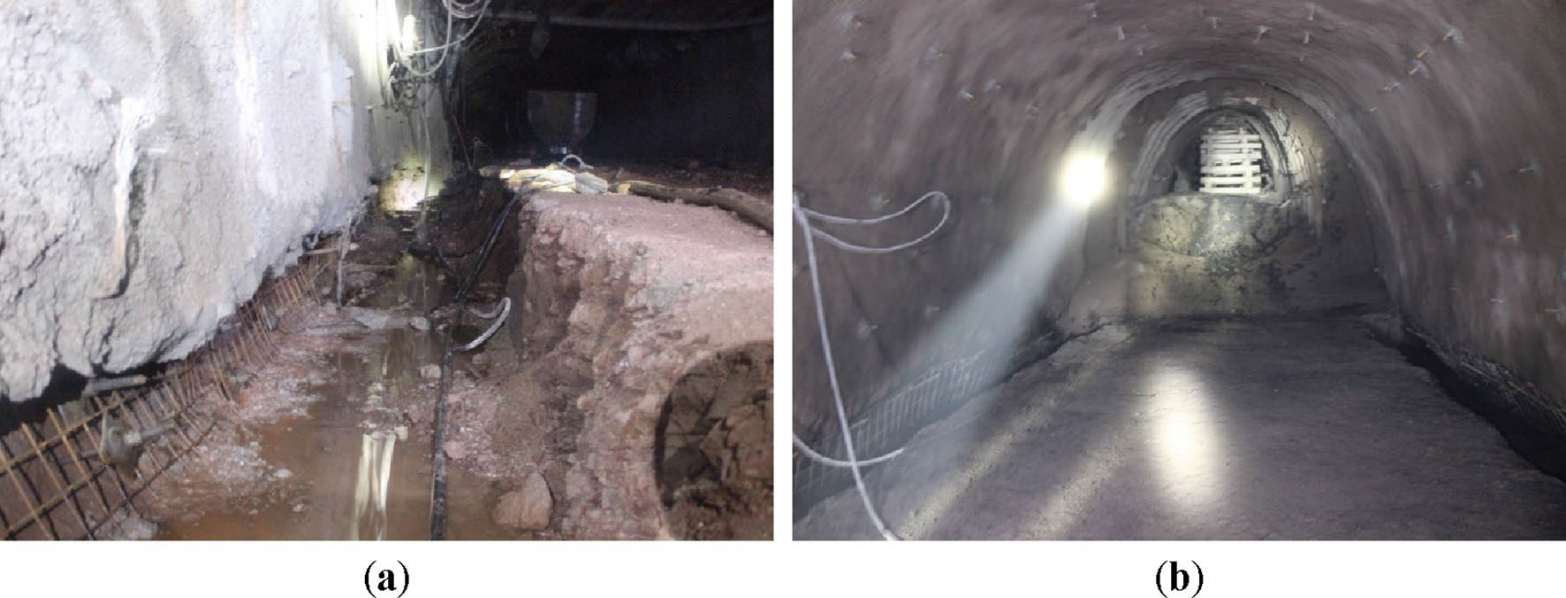
Renovation of the main stone gate of Qianjiaying Mine -850m. (**a**)Under construction. (**b**)After construction.

In order to test the safety and reliability of the optimized supporting parameters, the roof separation instrument was installed on the main crosscut at -850, and the displacement monitoring station was arranged by the "cross" point method to regularly observe and record the convergence and deformation of the roadway surrounding rock. After half a year of roadway surface convergence observation, it was found that the convergence of the two sides was up to 135 mm, and the angular displacement of the two shoulders was slightly larger. Compared with the previous support method, the displacement is reduced by about 35%, and the convergence of the roof and floor is within 75–115 mm. It can be seen that the support scheme of "three-level anchor bolt + bottom corner pressure relief + two-level anchor injection" can better realize the convergence control of the shallow displacement. The construction and application are shown in Fig. 12.

## Conclusion

1. XRD test shows that the clay mineral content of this type of surrounding rock is about 53–75%, mainly montmorillonite and kaolinite; SEM test shows that the microcracks of this type of surrounding rock are relatively developed. The internal mechanism of roadway deformation and instability is that the surrounding rock with high clay mineral content expands along the macro and meso cracks by hydration under the action of deep excavation stress, thus leading to the weakening and instability of the original anchorage structure.
2. Through the compressive test of wet and dry rock samples under different confining pressures, it is found that the softening effect of water on specimens with low confining pressures is extremely significant. The softening coefficient of uniaxial compressive strength is 63.1%, and the softening resistance is extremely poor. The surrounding rock should be modified by grouting reinforcement, so as to strengthen the bearing capacity in the anchoring area.
3. Based on the above test results, a collaborative control scheme of grouting and pressure relief is proposed and successfully applied in the field. The field engineering practice shows that the excavation of pressure relief grooving at two bottom corners of the roadway increases the allowable value of the deformation space in the early stage of the roadway, releases the expansion stress in the shallow loose area quickly through the pressure relief grooving, fills the primary and excavated fissure with multi-level grouting, blocks the expansion softening effect of water on the rock mass, and has a significant effect on controlling the deformation and instability of this kind of soft rock roadway.

## Data Availability

The data used to support the findings of this study are available from the corresponding author upon request.
